# Preparing for the Next Epidemic with Basic Virology

**DOI:** 10.1371/journal.ppat.1005182

**Published:** 2015-10-22

**Authors:** Michael S. Diamond

**Affiliations:** 1 Department of Molecular Microbiology, Washington University School of Medicine, St. Louis, Missouri, United States of America; 2 Department of Medicine, Washington University School of Medicine, St. Louis, Missouri, United States of America; 3 Department of Pathology & Immunology, Washington University School of Medicine, St. Louis, Missouri, United States of America

In my career, I have found that obtaining fundamental insights into how one virus “works” enables the study of other viruses, which ultimately has allowed my group to investigate basic principles of viral pathogenesis and host immune restriction. A running joke among members of my laboratory is that I have never met a virus I did not like, and the more exotic the name, the more excited I am. I confess this may be true. This practice has allowed me to collect and begin study of esoteric viruses that might or might not be the next plague (e.g., Usutu virus, Gadgets Gully virus, Barmah Forest virus, and O’nyong’nyong virus, to name a few). Why do this?

Our scientific training and experiences shape us. As an MD-PhD student in the late 1980s and early 1990s in Timothy Springer’s laboratory, I investigated the functions of two leukocyte adhesion receptors called Mac-1 (CD11b/CD18) and ICAM-1 (CD54). I recall the excitement of defining new adhesion molecule–receptor interactions and their roles in regulating immune cell function. The impact of these receptors was highlighted by the infections acquired by the rare people who genetically lacked functional proteins. At the time, I was struck by how quickly basic biology discoveries could be translated into therapeutic strategies. It seemed like every immunology faculty member had his or her own biotech company on the side, based on fundamental research from their laboratories! Since then, targeted antagonists of adhesion molecules indeed have become successful drugs for multiple sclerosis, coronary artery disease, and vascular disease.

Because of this relationship between immunology and human disease and a long-standing interest in global health, I chose infectious diseases for subspecialty training. In the late 1990s, as a postdoctoral fellow in Eva Harris’ laboratory, I began working at the bench in virology by studying virus–host cell interactions of Dengue virus (DENV), a mosquito-transmitted flavivirus responsible for considerable (and growing!) morbidity on a global scale. Beyond learning molecular virology and fundamental principles of the viral life cycle in the host, I became interested in defining mechanisms of host immune restriction, viral immune evasion, and viral pathogenesis.

As I began searching for a faculty position, a career-changing event occurred: West Nile virus (WNV), a related flavivirus, entered into the United States and caused disease in humans and other animals. In 2001, I decided to work on WNV and put on hold my studies of DENV innate immune evasion mechanisms, even though I had secured NIH support for them (I used the funding instead to study WNV pathogenesis—I hope the NIH has forgiven me for this). I made this career decision for several reasons: (1) WNV had public health implications, and there were few scientists studying it in 2001. I assumed (which fortunately came true) that the virus would not go away within a year; (2) I reasoned that if I knew enough biology about one virus, I could apply this knowledge to study related viruses; (3) I wanted to distinguish myself from my PhD mentor, who was an emerging leader in the Dengue field; (4) I was encouraged strongly by my faculty colleagues at Washington University to study something important even though I lacked experience in critical experimental areas (e.g., animal work). This is how I began developing a mouse model of WNV infection and investigating immune mechanisms of control. Using a series of genetically engineered knock-out mice that I received from many colleagues and other approaches, my laboratory defined, over a period of ten years, how B cells, CD4^+^ T cells, CD8^+^ T cells, complement, and type I and II interferons can restrict West Nile virus pathogenesis.

As my laboratory become productive, something else happened. I learned how exciting it was to collaborate on a project with your friends in the field. Collaborations allowed the pace of our discoveries to accelerate and the use of more inter-disciplinary structural, biochemical, genetic, and immunological approaches to address unanswered questions. With my colleagues in academia and industry, my laboratory has studied basic questions, including how antibodies engage and neutralize viruses, how innate immune genes control viral infection in cells, and how viruses can evade host immune responses to promote virulence. This study of basic flavivirus biology has informed the development (by us and others) of diagnostic tests, novel vaccine candidates, antibody-based therapeutics, and analyses of immune correlates of protection.

Which gets us back to the rationale for my collecting viruses. After seeing how the basic knowledge of one virus (DENV) enabled rapid progress on a related emerging virus (WNV) with implications for translational discovery, I became interested in studying many viruses. I did this for several reasons: (i) By having different viruses in the laboratory, we can test the broad significance of any given viral phenotype. This has been relevant for discerning the mechanism by which innate immune genes (e.g., IFIT and IFITM genes) restrict viral infection and interrupt pathogenesis. (ii) We can develop expertise with viruses from other families that are emerging. This has allowed us to pursue basic questions on the pathogenesis of positive-stranded alphaviruses (e.g., Chikungunya and Venezuelan equine encephalitis viruses) and negative-stranded bunyaviruses (La Crosse and Oropouche viruses). And (iii) should a new virus from these families emerge (for instance, Zika virus), we have the facility to begin studies quickly to address gaps in basic knowledge.

I believe most of my colleagues would agree that although we cannot always predict the next major viral epidemic, if we know enough fundamental virology (and biology), we can respond quickly to ask the right questions and perform the key experiments that allow for the expedited development of disease models, diagnostics, therapeutics, and vaccines. In my opinion, transformative and translational science happens on a foundation of basic research, and we need a profound knowledge base using model systems to gain an adequate breadth and depth of understanding. Preparedness will come not from spending millions of dollars on funding translational initiatives for what we think might be the next pandemic, but rather in investing more substantively in fundamental studies that make discoveries that can be applied to all pathogens.

**Image 1:**
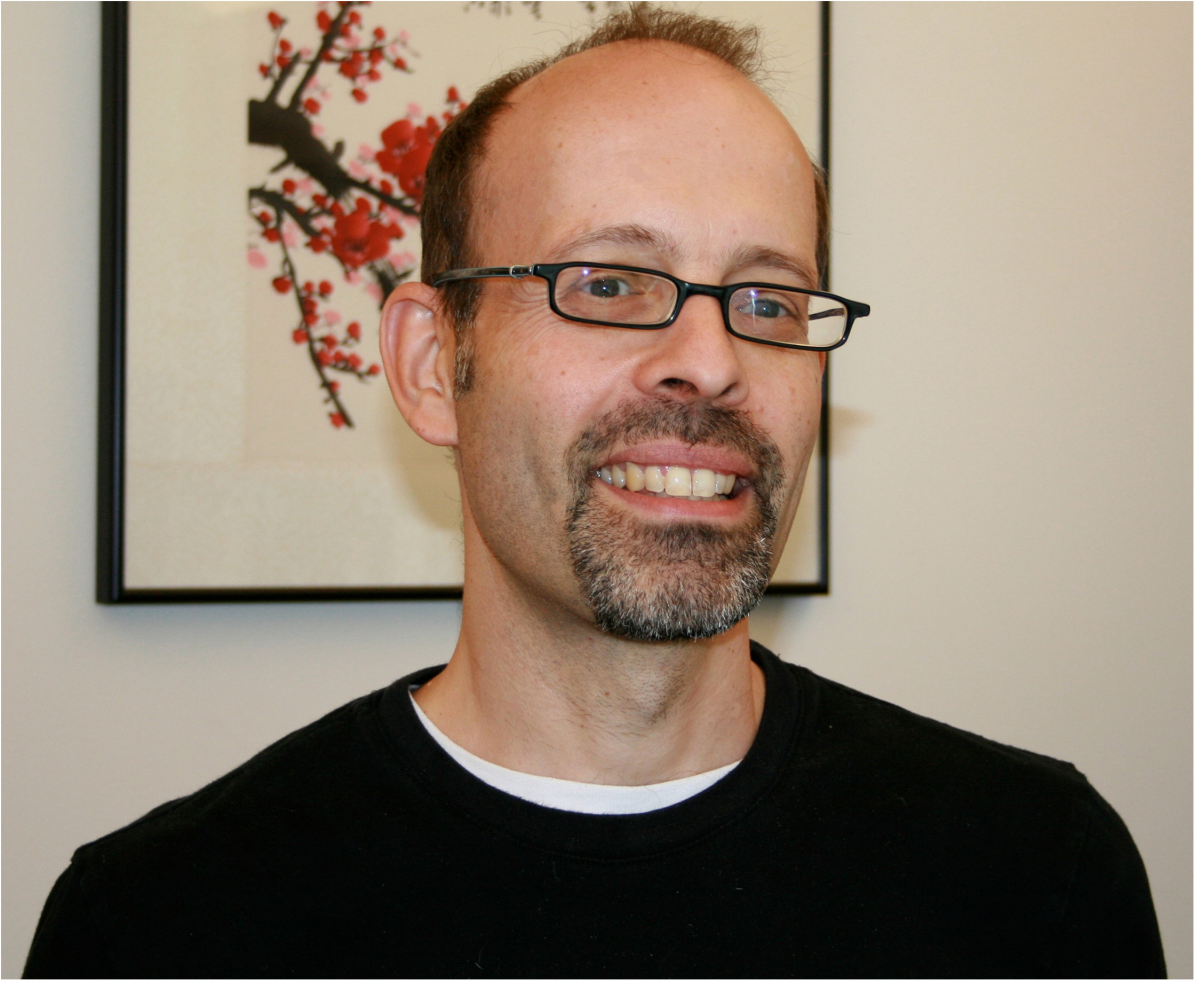
Michael S. Diamond.

